# Mutation spectrum of *PAX6* in Chinese patients with aniridia

**Published:** 2011-08-11

**Authors:** Xiaohui Zhang, Panfeng Wang, Shiqiang Li, Xueshan Xiao, Xiangming Guo, Qingjiong Zhang

**Affiliations:** State Key Laboratory of Ophthalmology, Zhongshan Ophthalmic Center, Sun Yat-sen University, Guangzhou 510060, China

## Abstract

**Purpose:**

To identify mutations in the paired box 6 (*PAX6*) gene of 33 probands with aniridia and to reveal the mutational spectrum in the Chinese population.

**Methods:**

Unrelated probands with aniridia from 27 newly selected families and six previously analyzed families participated in this study. The coding regions of *PAX6* in the 27 new families were analyzed using cycle sequencing. Families that lacked detectable variations based on sequencing (14 new and six previously analyzed) were further analyzed using multiplex ligation-dependent probe amplification (MLPA).

**Results:**

Fifteen mutations were identified in 16 of the 33 families: c.[65_94del30; 99_105dup7], c.101_102insA, c.177delG, c.238_239insGCGA, c.1033–42_1033–26del17insG, c.1A>G, c.120C>A, c.718C>T, c.949C>T, c.1062C>A, c.1183G>A, c.1268A>T, and three gross deletions involving exons 1–14, exons 8–14, and exons 9–14. The first five mutations were novel and the c.1268A>T mutation was present in two families. Phenotypic variations were observed between families and between different affected patients within the families.

**Conclusions:**

The *PAX6* mutation spectrum in Chinese aniridia patients is comparable to that reported in other ethnic groups. Further studies of the 17 families with no detected mutations may provide additional information to improve the understanding of the molecular genetics of aniridia.

## Introduction

Aniridia (OMIM 106210) is a bilateral, panocular disorder characterized by the absence of the iris. It is usually accompanied by developmental defects of the cornea, lens, retina, optic nerve, and/or the anterior chamber angle [1]. Approximately two thirds of all cases are transmitted as an autosomal dominant trait among families. The remaining one third are sporadic, with no family history [1-3]. Mutations in *PAX6* (OMIM 607108) have been shown to be responsible for aniridia in most patients [4-10]. In rare cases, *PAX6* mutations may cause other ocular abnormalities, such as cataracts [11], Peters anomaly [12,13], foveal dysplasia, microphthalmia, and optic nerve malformations [14,15]. To date, at least 334 mutations in PAX6 have been identified [16]. However, the absence of *PAX6* mutations in some cases of aniridia implies that mutations in additional genes cannot be excluded [6-10]. This possibility is supported by recent reports: 1) mutations in the forkhead box C1 gene (*FOXC1*, OMIM 601090) are associated with aniridia in two families [17,18] and 2) aniridia in patients with preserved visual function are not related to *PAX6* mutations [19].

Understanding the mutation spectrum and frequency will not only provide biomarkers for clinical practice but will also represent a fundamental basis for identifying any additional causative genes. The spectrum and frequency of *PAX6* mutations in Chinese aniridia patients has not been well identified for the following reasons [20-27]: 1) most reports only involved a single or a limited number of families and 2) mutations were usually detected by cycle sequencing (detection of small variations), in which a larger deletion involving part or all of the gene could not be detected. Cycle sequencing combined with multiplex ligation-dependent probe amplification (MLPA) has been shown to effectively detect small mutations and large deletions in *PAX6* [10]. In this study, both cycle sequencing and MLPA were used to detect mutations in the *PAX6* gene of 33 Chinese probands with aniridia.

## Methods

### Patients

Thirty-three unrelated probands with aniridia participated in this study, including six families previously analyzed by sequencing [22] and 27 newly selected families. Patients suspected of having Peters anomaly or Rieger syndrome were excluded. All aniridia patients in this study were recruited from our Pediatric and Genetic Eye Clinic at the Eye Hospital of the Zhongshan Ophthalmic Center, Guangzhou, China. Written informed consent that complied with the tenets of the Declaration of Helsinki and following the Guidance of Sample Collection of Human Genetic Diseases (863-Plan) by China’s Ministry of Public Health was obtained from each participant before the study. Genomic DNA was prepared from venous blood.

### Detection of *PAX6* mutations

Thirteen pairs of primers (Table 1) were used to amplify the 14 exons (3 noncoding exons and 11 coding exons) and their adjacent intronic sequences of *PAX6* (NCBI human genome build 37.2, NC_000011.9 for gDNA, NM_001604.4 for cDNA, and NP_001595.2 for protein). The PCR products of individual exons for each patient were sequenced using the ABI BigDye Terminator v3.1 Cycle Sequencing Kit (ABI Applied Biosystems, Foster City, CA) and the ABI 3100 Genetic Analyzer (ABI Applied Biosystems) according to the manufacturer’s recommendations. Sequencing results from patients’ sequences and *PAX6* consensus sequences from the National Center for Biotechnology Information (NCBI) human genome database (NC_000011.9) were imported into the SeqManII program of the Lasergene package (DNAStar Inc., Madison, WI) and aligned to identify variations. Each mutation was confirmed by bidirectional sequencing. Mutation descriptions followed the nomenclature recommended by the Human Genomic Variation Society (HGVS) [28].

**Table 1:** Primers used for amplification and sequencing of *PAX6*.

**Primer ID**	**Sequence (5′-3′)**	**Product length (bp)**	**Annealing Temperature (°C)**
Extra-E1-F*	GAGCTGTGCCCAACTCTAGC		
Extra E1-R	TCCATCTTTGTATGCCTCCTTT	399	56
Exon1F	GGAGAGGGAGCATCCAATC		
Exon1R	TCCTGGGAAGGAGACAGAGA	396	56
Exon2F	ACACACTTGAGCCATCACCA		
Exon2R	CTCCTGCGTGGAAACTTCTC	467	56
Exon3F	AGAGAGCCCATGGACGTATG		
exon3R	CCCAATCTGTTTCCCCTACA	318	56
Exon4F	TGCAGCTGCCCGAGGATTA		
Exon4R	GCACCCCGAGCCCGAAGTC	144	66
Exon5F	TCCCTCTTCTTCCTCTTCACT		
Exon5R	GGGGTCCATAATTAGCATC	301	61
Exon6–7F#	GCTCTCTACAGTAAGTTCTC		
Exon6–7R	AGGAGAGAGCATTGGGCTTA	457	61
Exon8F	GATTTGCAGGTGTCATCAAT		
Exon8R	ATATGGAGAGCTGCGTGGAT	212	65
Exon9F	TTTGGTGAGGCTGTCGGGA		
Exon9R	TCTTTGTACTGAAGATGTGGC	339	58
Exon10F	GTAGTTCTGGCACAATATGG		
Exon10R	GTACTCTGTACAAGCACCTC	206	62
Exon11–12F	GGCTCGACGTAGACACAGT		
Exon11–12R	TGCAGACACAGCCAATGAGG	500	62
Exon13F	GCTGTGTGATGTGTTCCTCA		
Exon13R	AAGAGAGATCGCCTCTGTG	245	62
Exon14F	CATGTCTGTTTCTCAAAGGG		
Exon14R	CCATAGTCACTGACTGAATTAACAC	202	61

^#^Exon numbers are based on the current version of gene structure and NM_001604.4, where the original exon 5a is numbered as exon 6. *This extra exon is based on another transcript variant NM_001127612.1, which is not present in the trancript variant of NM_001604.4.

### MLPA analysis

For patients who were determined not to have a *PAX6* mutation based on sequencing analysis, MLPA was used to detect deletions of part or all of *PAX6*, according to the manufacturer’s instructions (SALSA MLPA Kits P219-B1 *PAX6*; MRC-Holland bv, Amsterdam, the Netherlands) [10]. Briefly, 100 ng DNA samples were denatured for 5 min at 98 °C and then cooled to 25 °C. Probes were mixed and hybridized with DNA samples at 60 °C overnight and were then reacted with ligase 65 at 54 °C for 15 min, followed by 5 min at 98 °C and then held at 4 °C. Finally, all probes and sample ligations were amplified by PCR using specific carboxyfluorescein (FAM) labeled PCR primers. PCR products were separated by electrophoresis using the ABI PRISM 3100 Analyzer. Data analysis was performed using GeneMarker V1.5 software. A peak area between 0.7 and 1.3 times was considered normal; however, peak areas below 0.7 represent deletions and those above 1.3 represent duplications.

## Results

**Figure 1:**
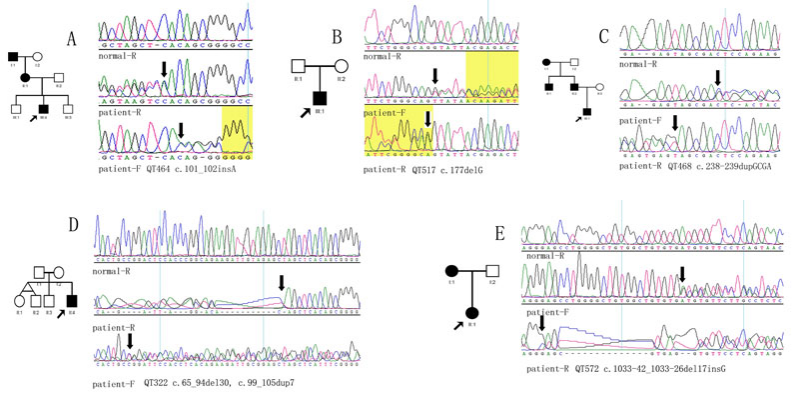
Frameshift mutations detected in *PAX6*. Five novel deletion/insertion mutations were identified in five probands with aniridia from unrelated families. Pedigrees (left) are accompanied with sequence chromatography (right). Arrows indicate the probands. R represents reverse sequence.

**Figure 2:**
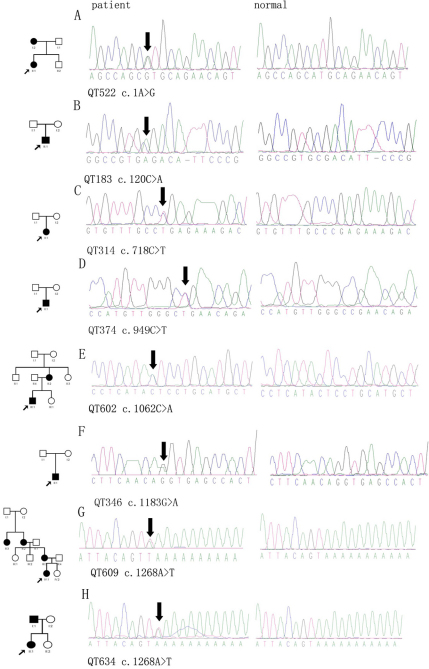
Point mutations detected in *PAX6*. One missense and six nonsense mutations in PAX6 were found in eight probands with aniridia from unrelated families. From left to the right, the columns represent pedigrees, sequencing results from probands with aniridia, and corresponding sequences from normal controls.

Sequencing of the 14 exons of *PAX6* of the 27 probands revealed 12 mutations in 13 patients, including five novel deletion/insertion mutations and seven known point mutations, as follows: c.[65_94del30; 99_105dup7] (p.P22LfsX25), c.101_102insA (p.Q34fsx21), c.177delG (R59SfsX19), c.238_239insGCGA (p.S80fsx12), c.1033–42_1033–26del17insG (splice change), c.1A>G (Initiation codon abolished), c.120C>A (p.C40X), c.718C>T (p.R240X), c.949C>T (p.R317X), c.1062C>A (p.Y354X), c.1183G>A (p.G395R), and c.1268A>T (p.X423LeuextX*15; Table 2, Figure 1 and Figure 2). Of the 12 observed mutations, eight resulted in a premature stop codon. The other four were involved in the splicing acceptor, initial codon, or the stop codon. No putative mutations were detected in the other 14 probands. No variants were detected in the three noncoding exons (exons 1~3).

**Table 2:** Clinical data and *PAX6* mutations in the 16 probands.

ID	Age (years)	Gender	Inheritance	Visual acuity	Clinical manifestation^#^			Mutations detected in *PAX6*		
					Cornea	Iris	Lens	Exon	Sequence variations	Effect
QT183	8	M	sporadic	0.1; 0.1	normal	complete aniridia	inferior opacity; upward dislocation	E5	c.120C>A	C40X
QT314	3/12	F	sporadic	NA	normal	complete aniridia	transparent	E9	c.718C>T	R240X
QT322	12	M	AD	0.1; 0.1	normal	subtotal aniridia	punctate opacity	E5	c.65_94del30.c.99_105dup7	P22LfsX25
QT346	3	M	sporadic	NA	inferior leucoma	complete aniridia	transparent	E12	c.1183G>A	G395R
QT350	4	M	sporadic	0.1; 0.1	normal	complete aniridia	transparent	E9–14	E9–14 del.	One copy deletion
QT374	7	M	sporadic	0.1; 0.1	normal	complete aniridia	transparent	E10	c.949C>T	R317X
QT462	4	F	sporadic	NA	normal	complete aniridia	transparent	E8–14	E8–14 del.	One copy deletion
QT464	19	M	AD	0.2; 0.2	normal	complete aniridia	punctate opacity	E5	c.101_102insA	p.H34QfsX21
QT467	3	F	AD	NA	normal	subtotal aniridia	punctate opacity	E1–14	E1–14 del.	One copy deletion
QT468	8	M	AD	0.2; 0.2	normal	circumpupillary aplasia	punctate opacity	E6	c.238_239dupGCGA	T80Sfsx12
QT517	2/12	M	sporadic	NA	normal	complete aniridia	transparent	E6	c.177delG	R59SfsX19
QT522	18	F	AD	0.1; 0.1	microcornea	complete aniridia	punctate opacity	E4	c.1A>G	Initiation codon abolished
QT572	17	F	AD	0.2; 0.3	normal	complete aniridia	punctate opacity	E12	c.1033–42_1033–26del17insG	Splice change
QT602	14	M	AD	0.1; 0.1	normal	circumpupillary aplasia	transparent	E12	c.1062C>A	Y354X
QT609	5	F	AD	0.1; 0.2	normal	complete aniridia	transparent	E13	c.1268A>T	X423LeuextX*15
QT634	7	F	AD	0.1; 0.1	normal	full iris*	punctate opacity	E13	c.1268A>T	X423LeuextX*15

Note: # Nystagmus and foveal hypoplasia were present in all patients with PAX6 mutations. AD: Autosomal dominant. NA: Not available. *Her father had the same mutation but had complete aniridia.

**Figure 3:**
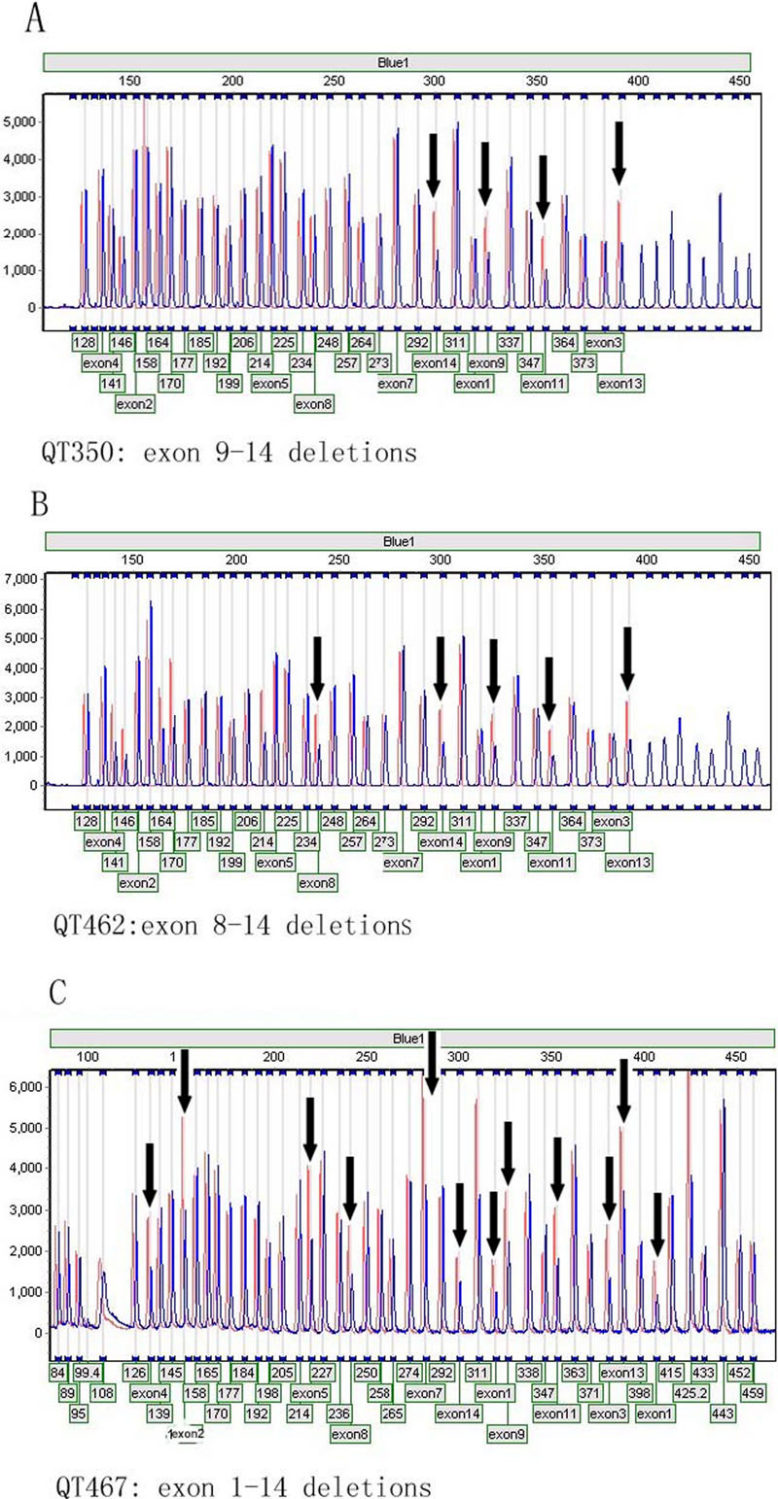
*PAX6* mutations detected by MLPA. Three gross deletions were involved in exons 9–14, 8–14, and 1–14, respectively. Black arrows indicate the exons with deletions, in which each peak area is below 0.7 compared to internal controls.

For the 20 probands in whom *PAX6* mutations were not detected by sequencing, including the remaining 14 probands from this study and six probands from our previous study [22], we detected *PAX6* deletions involving multiple exons (exons 1–14, 8–14, or 9–14, respectively) in three probands detected by MLPA (Table 2, Figure 3).

**Figure 4:**
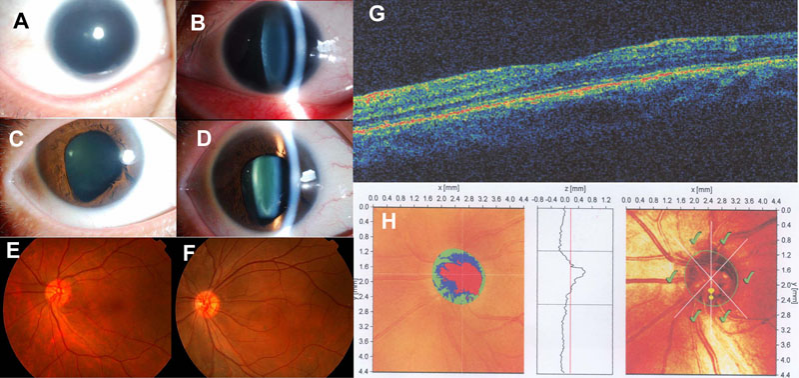
Photos showing the clinical phenotype of patients with a heterozygous c.1A>G mutation in *PAX6*. **A** and **B**: Complete absence of iris and microcornea (9.5 mm in diameter) was observed in both eyes of the proband, an 18-year-old girl. **C** and **D**: A partial defect of the bilateral iris mimicking iris coloboma was present in the proband’s mother, who also had the mutation. **E** and **F**: Foveal hypoplasia was observed in the mother (**E**) and the proband (**F**). **G** and **H**: A flat fovea (**G**) and optic disc hypoplasia (**H**) in the mother were demonstrated by optical coherence tomography and Heidelberg retinal tomography, respectively.

Of the 16 probands with *PAX6* mutations, all had congenital nystagmus and foveal dysplasia. Complete aniridia, subtotal aniridia, circumpupillary aplasia, or a full iris in both eyes was recorded in probands 11, 2, 2, and 1, respectively (Table 2). One proband (QT634) had a full iris, but his father harbored the same heterozygous *PAX6* mutation and had complete aniridia. Additional ocular abnormalities included lens opacities (eight probands), inferior corneal leukoma (one proband with the missense mutation), and microcornea (one proband). The visual acuity of all probands ranged from 0.1 to 0.3, except for five children who were too young to be evaluated (Table 2). Phenotypic variations in the family harboring the c.1A>G mutation were observed: one proband (QT522) had complete absence of the iris in both eyes, although her mother with the same *PAX6* mutation only had iris defects similar to iris coloboma (Figure 4). The patient with the missense mutation (c.1183G>A, p.G395R) had typical aniridia and inferior corneal leukoma.

## Discussion

The mutation frequency of *PAX6* in Chinese aniridia patients is similar to that in Caucasian aniridia patients. In this study, *PAX6* mutations were identified in 16 of the 33 families tested. When the results of this study are combined with those of our previous study [22], the *PAX6* mutations have been identified in 21 of 38 unrelated patients using cycle sequencing and MLPA. Of the 21 patients, mutations in 18 patients were identified by analyzing *PAX6* coding regions using direct sequencing, and mutations in 3 patients were detected using MLPA. For *PAX6* mutations in Chinese aniridia patients, the overall mutation detection rates detected with cycle sequencing, MLPA, or both were 47% (18/38), 8% (3/38), and 55% (21/38), respectively. In a similar study of Caucasian aniridia patients [10], *PAX6* mutations were detected in 49% (34/70), 11% (8/70), and 60% (42/70) patients with cycle sequencing, MLPA, or both, respectively. Several other studies have detected aniridia-associated *PAX6* mutations in 30% (9/30) of Mexican patients [29], 56% of Indian patients [30], 67% (4/6) of Thai patients [31], 38%–58% (3/8–14/24) of German patients [32,33], 50% (2/4) of Japanese patients [34], 79% (30/38) of Danish patients [35], and 83%–94% (10/12–67/71) of British patients [9,36]. The detection of these mutations was based solely on sequence analysis in most studies, but chromosomal analysis was additionally performed in a few studies. These reports demonstrate that while *PAX6* mutations were prevalent, they were not detected in all patients with aniridia. One reason that may account for this is the possibility that small variations outside the exons, such as intronic regions [10], may not be detectable by the techniques used to analyze *PAX6*. Furthermore, frequent chromosomal rearrangements have been described in aniridia patients previously [37], which may not be detectable by cycle sequencing and MLPA. It is also possible that there are mutations in other genes which contribute to aniridia given that mutations in *FOXC1* are associated with aniridia [17,18] and that no *PAX6* mutations were detected in aniridia patients with preserved visual function [19].

The spectrum of *PAX6* mutations in aniridia is similar within both Chinese and Caucasian patient cohorts. The majority of *PAX6* mutations reported so far would lead to truncation of encoded proteins (such as nonsense, splicing, insertion, or deletion mutation) and only about 2%–11.7% are missense mutations [38,39]. In one review [38], 257 aniridia-associated mutations were classified as nonsense mutations (38.9%), splice mutations (13.2%), frame-shifting insertions or deletions (25.3%), in-frame insertions or deletions (6.2%), missense mutations (11.7%), and run-on mutations (4.7%). For the 21 mutations in the Chinese patients analyzed in the present study, these percentages are 33.3%, 14.3%, 19.0%, zero, 9.5%, and 9.5%, with an additional 14.3% being gross deletions of the *PAX6* gene. In addition, seven point mutations detected in this study are known mutations, suggesting common mutations. Of the seven, the p.R240X and p.R317X mutations involving CpG dinucleotides are the most common nonsense mutations in *PAX6*. The p.C40X mutation was detected in one patient in this study and another patient in our previous study [22]. The run-on mutation, X423LeuextX*15, was detected in two unrelated Chinese patients.

In this study, we detected five novel small insertion/deletion mutations, seven known point mutations, and three known gross deletions in 33 unrelated aniridia patients. In this and one of our previous studies [22], the *PAX6* gene was analyzed by cycle sequencing and MLPA in 38 unrelated aniridia patients. However, *PAX6* mutations were only detected in 55% (21/38) patients. Further studies of the 17 families without *PAX6* mutations may elucidate the molecular basis of aniridia in these families.
